# A comparison of traditional anterior colporrhaphy, polypropylene mesh and porcine dermis in cystocele repair

**Published:** 2007

**Authors:** Pratipal Singh, Manu Gupta, Aneesh Srivastava

**Affiliations:** Department of Urology and Renal Transplantation, Sanjay Gandhi Post Graduate Institute of Medical Sciences, Lucknow, India. E-mail: aneesh@sgpgi.ac.in

## SUMMARY

This is a retrospective review of 119 patients who underwent cystocele repair by the same urologist using porcine dermal graft, polypropylene mesh or traditional repair from January 1999 to August 2005. Average follow-up of 13.5 months (range two to 46) was available for 99 patients. Fifty-six (57%) underwent cystocele repair using porcine dermal graft, 25 (25%) received polypropylene mesh and 18 (18%) underwent traditional repair. Twenty-two (22%) patients had cystocele recurrence. Thirty-six per cent patients (20 of 56) with porcine dermal grafts had recurrence compared to 4% (one of 25) and 6% (one of 18) using polypropylene and traditional repair, respectively. Mean time to cystocele recurrence was 4.9 months (range 0.5 to 20). Twelve patients (21%) had extrusion of porcine grafts through the anterior vaginal wall incision compared to one (4%) with polypropylene mesh. Short-term failure rate for anterior vaginal wall prolapse using porcine dermis interposition graft was higher than that for traditional anterior colporrhaphy or polypropylene mesh. In addition, the incidence of vaginal extrusion of porcine graft was unacceptably high. Porcine dermis is a less suitable material for cystocele repair than polypropylene mesh or traditional anterior colporrhaphy.

## COMMENTS

Recurrent anterior vaginal wall prolapse can develop in more than 20% of patients undergoing traditional anterior colporrhaphy. In light of this high recurrence rate many surgeons have been incorporating synthetic or allograft mesh to augment the repair.

In this study there are some shortcomings, including retrospective design, short follow-up for a study spanning almost six years and a lack of validated questionnaires. Authors report a negative experience with porcine dermis, both in terms of success and extrusion rate. However, others have reported better outcome[1] which may be due to different follow-up times and outcome measures. Apart from this, biological grafts are also associated with allergic reactions and disease transmission. There is paucity of data regarding prospective randomized trials on the use of these biomaterials prior to their widespread human need which might help in reducing such type of experiences. Unless such data are available surgeons should give serious thought prior to embarking on the use of such biological materials.[2]
